# Effects of Caffeine, a DNA Damage Response Inhibitor, on Papillomavirus Genome Replication

**DOI:** 10.3390/pathogens11111298

**Published:** 2022-11-05

**Authors:** Sriramana Kanginakudru, Timra Gilson, Leny Jose, Elliot J. Androphy

**Affiliations:** 1 Department of Dermatology, Indiana University School of Medicine, Indianapolis, IN 46202, USA; 2 Department of Microbiology and Immunology, Indiana University School of Medicine, Indianapolis, IN 46202, USA

**Keywords:** HPV, caffeine, replication

## Abstract

Epidemiological studies have revealed that caffeinated coffee imparts a reduced risk of oropharyngeal cancer, of which human papillomavirus (HPV) is one of the causative agents. Caffeine is a known inhibitor of the DNA damage response (DDR) pathway. We sought to test the effects of caffeine on the early replication of the HPV31 virus. It has been reported that the inhibition of several factors necessary for the DDR during the differentiation-dependent stage of HPV block genome amplification, while the HPV genome maintenance replication was unaffected. We first studied the effects of caffeine in the earliest stages of viral infection. Using pseudo-virions (PsV) expressing an m-Cherry reporter gene and quasi-virions (QsV) containing HPV31 genomes to mediate the infection, we found no evidence that caffeine impeded the viral entry; however, the infected cells displayed a reduced HPV copy number. In contrast, caffeine exposure increased the copy number of HPV31 episomes in the transient transfection assays and in the CIN612E cells that stably maintain viral episomes. There was a concomitant increase in the steady state levels of the HPV31 E1 and E2 transcripts, along with increased E2 loading at the viral origin of replication (ori). These results suggest that the caffeine-mediated inhibition of the DDR reduces viral genome replication in the early stage of infection, in contrast to the maintenance stage, in which the inhibition of the DDR may lead to an increase in viral amplicon replication.

## 1. Introduction

Signaling pathways that are activated upon encountering damaged DNA, collectively known as the DDR pathway, allow the cell to block or, alternatively, allow virus replication. Viruses such as adenovirus, polyomavirus, and HPV have evolved mechanisms to either utilize or inhibit the DDR for their sustenance [1,2]. HPV, which are non-enveloped, small, double-stranded DNA viruses that are associated with common cutaneous warts, as well as cervical and oropharyngeal cancers, induces the DDR during their replication cycle [3,4]. The activation of the DDR has been attributed to the PV E7 protein and E1 DNA helicase [5,6]. RNAi depletion or the chemical inhibition of the DDR factors nibrin (Nbs1), ataxia-telangiectasia mutated (ATM), ATM- and Rad3-related (ATR), and DNA topoisomerase 2-binding protein 1 (TopBP1) blocked HPV differentiation-dependent genome amplification, implying that the DDR mediates a necessary function for genome replication at this stage [7,8,9,10,11]. Intriguingly, E6 proteins of the beta classes HPV 5 and 8 genotypes abrogate ATR activation through p300 degradation [12]. HPV16 E6 was reported to deregulate the Checkpoint kinase 1 (CHK1), interfering with replication at stalled replication forks [13]. Recent evidence supports the roles of both the E6 and E7 of HPV 8 in the autophagic degradation of CHK1 [5], which can interfere with DDR activation. In in vitro transient replication assays in cell culture, E1- and E2-mediated replication continued in the presence of caffeine and wortmannin, which inhibit DNA-damage-responsive kinases, including ATM-, ATR-, and DNA-dependent protein kinase [14].

Epidemiological meta-analysis has shown an inverse association of caffeinated coffee with oropharyngeal cancer mortality [15]. In addition to tobacco and smoking, HPV is an established contributor to head and neck cancers. Caffeine, a plant alkaloid and main ingredient of coffee, is an inhibitor of TopBP1-mediated ATR activation [16]. Caffeine has several other pharmacological activities. In vitro experiments suggested its anti-hepatitis C virus activity, and a metanalysis inferred a reduced risk of liver fibrosis and inflammation in HCV patients who consume caffeine [17,18]. Due to its anti-inflammatory and immune modulation activities, caffeine is hypothesized to provide health benefits by interfering with virus entry and replication in SARS-CoV2 infection [19]. However, despite its repressive characteristics in regard to DDR and reduced mortality in oropharyngeal cancers, the effects of caffeine on early HPV DNA replication has not been documented. Here, we demonstrate that caffeine treatment reduces the early replication after HPV infection and, in contrast, increases the viral copy number in cells that maintain HPV episomes.

## 2. Results

### 2.1. Caffeine Reduces UV-Induced Cell Death and Phosphorylation of H2AX

**Figure 1:**
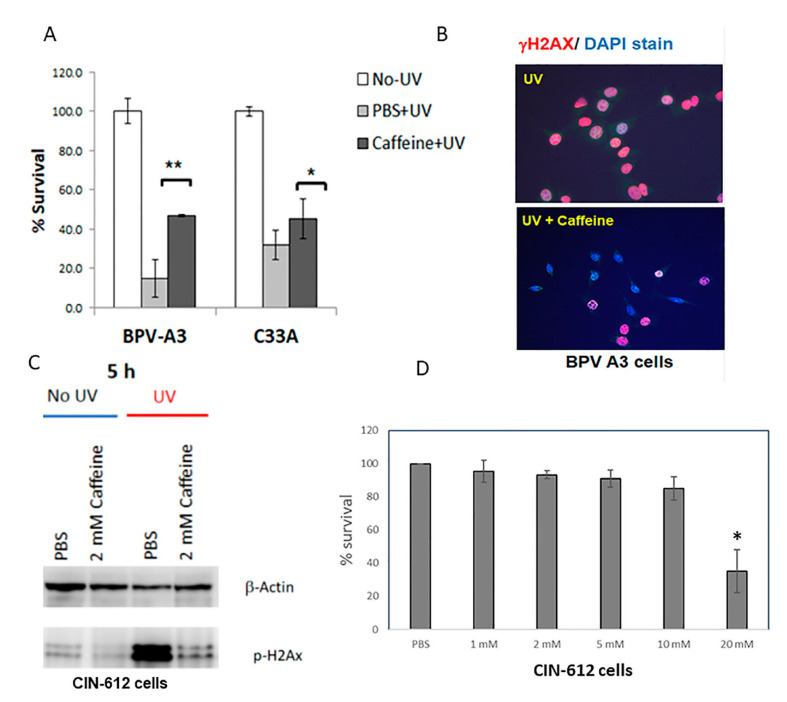
Caffeine reduced the induction of DDR in UV-irradiated cells: (**A**) C33a cells exposed to UV at 200 mJ/cm^2^ were protected against cell death by 2 mM caffeine. (* *p* < 0.05, ** *p* < 0.02). (**B**) BPV-A3 cells exposed to 200 mJ/cm^2^ displayed reduced H2AX phosphorylation after overnight treatment with 2 mM caffeine; blue—DAPI stain, red—γH2AX. (**C**): Caffeine reduced the UV-induced phosphorylation of H2AX in CIN612E cells. Cells were pre-incubated with 2 mM caffeine and exposed to 200 mJ/cm^2^ UV. Five h post-irradiation, Western blotting of γH2AX demonstrated reduced phosphorylation in caffeine-treated cells. (**D**) Survival of CIN612 cells treated with increasing concentrations of caffeine for 48 h. Average of 3 experiments * *p* < 0.05.

To test whether caffeine, a known inhibitor of ATM/ATR, promoted cell survival following DNA damage, C33a and bovine-papillomavirus-positive BPV-A3 cells were treated with 2 mM caffeine prior to exposure with 200 mJ/cm^2^ of UV light. After 24 h, the cell survival was measured using an MTS colorimetric assay. As shown in Figure 1A, caffeine significantly (*p* < 0.05) increased the survival after UV exposure by nearly threefold in the BPV-A3 cells that harbor episomal BPV-1 genomes, and in the p53 mutant C33a cells, the survival increased by 20%. The phosphorylation of chromatin protein H2AX at serine 139 (termed γH2AX) is an early response to DNA damage. The BPV-A3 cells showed a reduced phosphorylation of the H2AX protein after exposure to caffeine (Figure 1B), whereas in the case of the PBS control UV-irradiated cells, almost all the cells displayed γH2AX foci. These results confirm that caffeine increases cell survival and reduces the γH2AX formation elicited by UV-induced DNA damage. To further evidence the inhibitory effect of caffeine, CIN612E cells, which express episomal HPV-31 genomes, were UV-irradiated and lysates were blotted for γH2AX. The caffeine-treated cells showed decreased γH2AX compared to the controls at 5 h post-irradiation (Figure 1C), further establishing its ability to impede the activation of the DDR. To determine whether caffeine alone caused cytotoxicity in the CIN612 cells, we treated them with increasing concentrations of caffeine. At 20 mM, caffeine reduced the cell viability by 70%, as measured by calcein AM assay (Figure 1D).

### 2.2. Caffeine Affects Initial Amplification following Infection but Not Viral Entry

**Figure 2:**
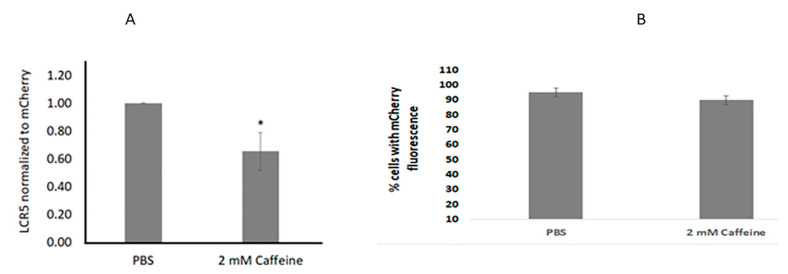
(**A**): Caffeine decreased the HPV31 copy number after infection. N/TERT cells were infected with both mCherry PsV and HPV31Neo QsV. DNA was extracted after 72 h, and the HPV31 copy number was measured by qPCR of HPV31 LCR and normalized to the non-replicating mCherry gene (* *p* < 0.05). (**B**): Cells were infected with mCherry PsV, and the percentage of fluorescent cells in the presence or absence of 2 mM caffeine was estimated after 72 h.

We sought to measure the effect of caffeine on viral genome establishment following infection and on virus entry. N/TERT cells were infected with a mixture of mCherry PsV and HPV31Neo QsV. We previously reported that cells can be simultaneously infected with different PsV using the SMI method [20]. SMI was performed in the presence or absence of caffeine. The HPV31 copy number was normalized to the non-replicating mCherry gene by qPCR. Beta actin was used as an internal control. The HPV31 genome copy number decreased by 40% in the 2 mM-caffeine-treated cells after 72 h compared to the PBS controls (Figure 2A). These results suggest that the initial amplification following infection is sensitive to caffeine and may depend on the activity of the DDR.

To test whether the reduction in the copy number was due to a decrease in the virus entry, we infected N/TERT cells with mCherry reporter PsVs alone. At 72 h post-infection, the red fluorescent cells were visualized and counted. There was no significant difference in the percentage of mCherry-expressing cells incubated in the media with caffeine (Figure 2B). From this experiment, we conclude that the initial uptake, as well as the m-cherry expression, were not affected by caffeine. Taken together with the results of the PsV-QsV dual infection, we propose that caffeine restricts the initial establishment of the HPV31 copy number by interfering with HPV replication following the virus entry.

**Figure 3:**
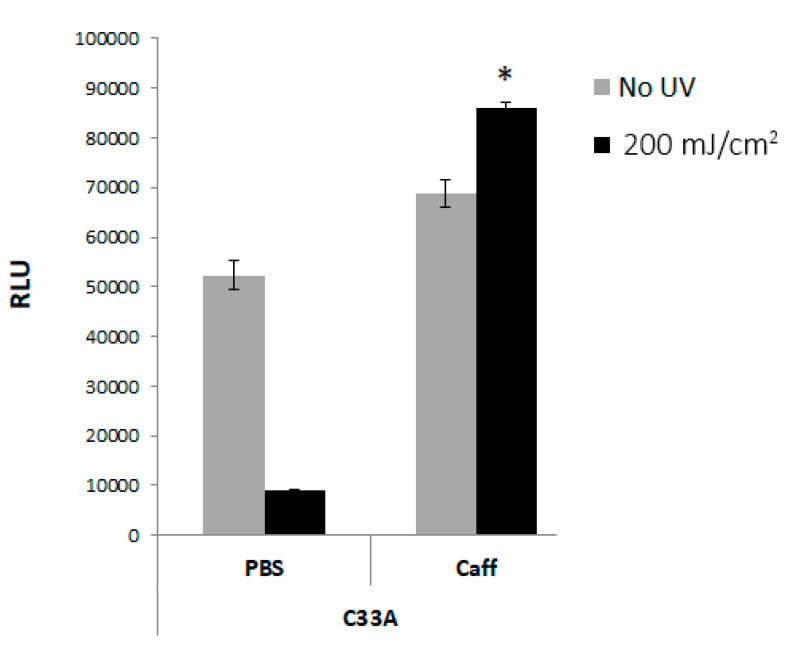
HPV31 replication continued after UV exposure in C33a cells that were treated with 2 mM caffeine, as measured by luciferase-based triple-plasmid assays. Caffeine-pretreated cells were transfected with plasmids and exposed to UV, and 48 h later, the luciferase activity was measured as relative light units (RLU) (* *p* < 0.05).

To test whether the blocking of the DDR pathway by caffeine in the UV-irradiated cells affected the virus replication, we used the HPV31-luciferase-based replication assay by transfection of the plasmids expressing E1 and E2, an ori-containing firefly luciferase reporter, and a non-replicable Renilla luciferase gene into the C33a cells. The cells were pretreated with 2 mM caffeine, transfected with the plasmids, and exposed to 200 mJ/cm^2^ of UV light. The luciferase activity, which reflects HPV replication, was measured after 48 h and was found to be increased by 8-fold in the caffeine-exposed irradiated cells compared to the control cells without caffeine (Figure 3. These results show that caffeine reduced the rate of cell death after the UV treatment and that the surviving cells continued to perform HPV DNA synthesis. A moderate and significant 20% increase in the replication rate was also observed in the caffeine-treated, foil-protected cells.

### 2.3. Caffeine Affects PV Episomal Maintenance

**Figure 4:**
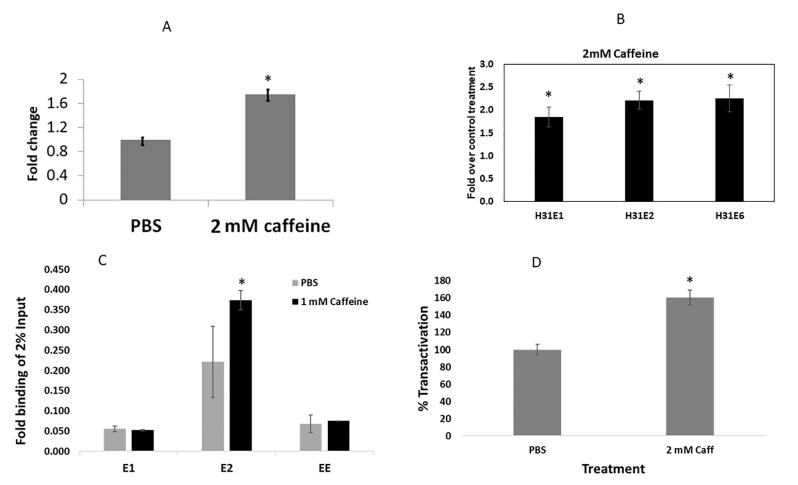
(**A**) Increased episomal copy number in CIN612E cells treated with 2 mM caffeine for 72 h. Realtime PCR of HPV31 LCR locus normalized to the β-actin gene showed a 1.8-fold increase with caffeine exposure (* *p* < 0.05). (**B**) HPV31 early gene transcripts were increased following 2 mM caffeine treatment for 72 h in CIN612E cells. RT-PCR of total RNA from CIN612E cells for the expression of 3 virus transcripts revealed increased transcripts. *X*-axis represents the HPV31 E1, E2, and E6 transcripts. (**C**) ChIP reveals increased loading of E2 at HPV31 LCR region in double-thymidine-blocked CIN612E that were cultured in the presence of 1 mM caffeine. *X*-axis represents the antibody used in the pulldown. * *p* < 0.05. (**D**): Caffeine increased transcription from an E2 binding site promoter in C33a cells transfected with a luciferase reporter gene and BPV-E2 following incubation with 2 mM caffeine (*p* < 0.05).

To test the effects of caffeine on the papillomavirus episomal maintenance, CIN612E cells were incubated with 2 mM caffeine for 72 h and lysed, and then total DNA was extracted. The HPV31 copy number was measured by quantitative real-time PCR using HPV-31-specific primers and normalized to genomic DNA using actin primers. Caffeine exposure increased the episomal copy number by 75% (Figure 4A), consistent with the transient replication assay results. Taken together, this suggested that caffeine increased the copy number through the transient increase in the virus replication. Since HPV DNA replication depends on the E1 and E2 viral proteins, we next sought to evaluate whether caffeine treatment affected the early mRNA transcripts. Total RNA was isolated from CIN612E cells, which were exposed to caffeine for 72 h, and the HPV31 E1, E2, and E6 transcript levels were measured by quantitative reverse-transcriptase PCR. Upon normalization to cellular actin mRNA, we observed 30, 45, and 40% increased E1, E2, and E6 transcripts, respectively (Figure 4B). Our results suggested that caffeine increased the viral transcription, which may be caused by an increase in the number of viral genome copies. To determine whether the elevated E1 and E2 transcripts observed after the caffeine treatment resulted in the augmented loading of these proteins at the HPV origin, chromatin immunoprecipitation (ChIP) of E1 and E2 was carried out on the CIN612E cells arrested in S phase. To prevent the combined toxicity of the thymidine and caffeine, the latter was reduced in the media to 1 mM. As shown in Figure 4C, the caffeine-treated cells displayed a 1.5-fold increased presence of HPV31-E2 at HPV ori compared to the PBS control. We did not detect a significant change in the HPV31-E1 loading. To address whether the caffeine altered the transcriptional activity of E2, we performed luciferase-based transient transcriptional assays with BPV E2, which is a strong transcriptional activator. Caffeine increased the BPV-E2 activity by 60% (Figure 4D).

## 3. Discussion

Papillomaviruses have a tri-phasic replication cycle in cell culture models. After PVs infect the basal epithelial cells, their genome undergoes initial establishment replication, in which a single-copy episome is amplified to perhaps tens of copies. After this initial establishment phase, the virus is maintained at a steady state of tens to hundreds of episomes as the cells divide. In the differentiation of keratinocytes, the viral genome undergoes a rapid expansion of the copy number by vegetative amplification. The proteins involved in the DNA damage response, including Nbs1, ATM, and ATR, are important for this amplification [7,8,9,10,11]. The roles of DDR factors in the initial amplification following the nuclear entry and maintenance phases have not been established. Caffeine is a nervous system stimulant found in very popular beverages, such as coffee, and has a vast repertoire of pharmacological and biochemical activities, including the inhibition of the DDR. Caffeine also appears to reduce the risk of developing oropharyngeal cancer, of which HPV is one of the causative agents. Here, we demonstrated that caffeine, at a concentration sufficient for the inhibition of the DDR pathway, augments HPV episome maintenance in CIN612E cells with endogenous HPV31 genomes. Upon treatment with caffeine, we observed an increased transcription of the early virus genes E1 and E2 and increased occupancy of E2 on the HPV ori. Using an E2-binding, luciferase-based construct, we also observed an enhancement of the BPV-E2-dependent transcriptional activity. We did not detect a significant change in the cell cycle distribution with a concentration of caffeine at or below 2 mM, which was the amount used in these experiments (data not shown). However, the cytotoxicity assays demonstrated that concentrations of caffeine of ≥20 mM induced significant cytotoxicity in the CIN612 cells. Caffeine was shown to inhibit SMC1 and ATR phosphorylation in CIN612 cells [21]. We showed that 2 mM caffeine effectively blocked the DDR and chose to use this concentration to reduce the potential off-target effects. Interestingly, a cup of coffee (250 mL) may contain 100 mg or about 2 mM caffeine, which becomes greatly diluted following ingestion. Correlation studies are required to indicate an association between coffee consumption and HPV persistence.

DNA double-strand break repair occurs through mechanisms such as homologous recombination (HR), non-homologous end joining (NHEJ), single-strand annealing (SSA) or microhomology-mediated end joining (MMEJ) (reviewed in [22]). HPV E7 was reported to promote MMEJ by suppressing NHEJ [23]. HR proteins are recruited for the stabilization of the replication fork (reviewed in [24]). Several HR factors are known to be engaged in papillomavirus amplification. Rad51, a principal mediator of HR that is also active in the DDR, is one of the established facilitators of HPV genome amplification [25]. Caffeine has a dual opposing effect on HR; it facilitates DNA synthesis after the initial strand invasion, but upon prolonged exposure, HR is inhibited by the depletion of key factors [26]. Furthermore, caffeine inhibits HR by promoting non-productive HR, likely by interfering with Rad51-mediated nucleofilament disruption [27]. These contrasting consequences of caffeine are likely concentration-dependent. In the present study, we primarily exposed cultured cells to 2 mM caffeine, which was generally well-tolerated and within the reported concentration range for the inhibition of ATM and ATR kinases [12,28]. To confirm this, we demonstrated that this amount of caffeine protected the cells from UV-induced death (Figure 1).

In some respects, the initial infection and establishment of virus episomes in conducive cells mimic the differentiation-dependent amplification. Multiple rounds of viral DNA replication must occur, in contrast to the once-per-cell-cycle genome replication that governs the host chromosomes. These two stages of amplification result in tens to thousands of copies per cell. However, unlike keratinocyte-differentiation-linked amplification, the role of the DDR upon the initial infection is unresolved. We tested the effect of caffeine in the initial 72 h after virus infection. By dual infection with PsV-QsV and by comparing the copy numbers of a non-replicating mCherry virus and replicating HPV31Neo QsV, we found that caffeine reduced the copy number of HPV31. The fluorescent microscopic observation of the mCherry-infected N/TERT cells revealed that caffeine did not compromise the virus entry, supporting our conclusion that HPV replication itself, and not virus entry, was affected by caffeine.

The crosstalk between genome replication and the cell’s DDR and HR pathways is beginning to be unraveled. Intricate interactions between different cellular and molecular pathways makes it challenging to pinpoint the precise target of caffeine during HPV replication. In this context, our observations, supporting the notion that caffeine has a dichotomous activity in papillomavirus replication, being inhibitory during early replication and stimulatory in the maintenance mode, may shed light on the differential mechanism of HPV genome replication during the early infection and episomal maintenance stages. Caffeine increased the levels of the E1 and E2 transcripts, which may account for the increased DNA replication. Studies using in vivo models to experimentally test the effects of caffeine on the HPV replicative program are warranted, as are epidemiologic studies of the relationship between caffeine and the infection, persistence, and malignant progression of HPV.

## 4. Materials and Methods

### 4.1. Cell Culture, Plasmids, and Antibody

C33a, an HPV-negative cell-line, BPV-A3, a mouse cell-line containing BPV mutant episomes, and 3T3-J2 and HEK-293TTF cells were maintained in DMEM medium supplemented with 10% FBS, 1X antibiotic mix (Invitrogen) at 37 °C and 5% CO_2_. CIN612E cells were grown in E-media with 3T3-J2 feeder cells [29]. N/TERT cells were grown in K-SFM media supplemented with bovine pituitary extract (Thermo-Fisher, City, Country) and antibiotics. The plasmids pBR322-HPV-31neo, pmCherry, Flag-31E2 used for the HPV31 luciferase reporter replication assays were described [29,30,31]. The HPV E1 and E2 antibodies were previously reported [31,32]. Anti-phospho-Histone H2A.X (Ser139) antibody was purchased from Millipore.

### 4.2. Luciferase-Based Transient Replication Assay

The luciferase-based replication assays of the C33a cells were essentially conducted as previously described [33], with a slight modification. Briefly, 80 ng each of the HPV-31 E1, E2, and pFLORI31 constructs was co-transfected along with *Renilla* luciferase at 16 ng per well on a 12-well plate. Twenty-four h post-transfection, the cells were exposed to 2 mM caffeine (dissolved in PBS; Sigma-Aldrich, St. Louis, USA) for the indicated times. The cells were lysed in Promega Dual-Glo luciferase reagent, and both the firefly and *Renilla* luciferase activity were determined using PHERAStar FS (BMG Labtech, Ortenberg, Germany). The firefly luciferase activity was normalized to the *Renilla* luciferase activity, and the value for the control was set as 100%.

### 4.3. UV-Induced DNA Damage Response—Cell Survival and Replication Assays

The C33a cells were seeded on 96-well plates and pretreated with 2 mM caffeine or PBS control overnight. The next day, half of the plate was wrapped with foil and was exposed to 200 mJ/cm^2^ UV in a Stratalinker 1800 UV Crosslinker (Stratagene, La Jolla, CA, USA). The cells were further incubated with or without caffeine. A cell survival assay was carried out using the CellTiter 96 AQueous One Solution Cell Proliferation Assay (MTS) kit (Promega, Madison, WI, USA) or calcein AM assay (Cayman Chemicals, Ann Arbor, MI, USA). The foil-covered cells were considered as 100% baseline for the absorbance determination. To model the DNA replication, the C33a cells were transfected with luciferase-based plasmids and treated with 2 mM caffeine. Then, 24 h later, the cells were exposed to 200 mJ/cm^2^ UV light and incubated for another 48 h, and then firefly luciferase assays were performed.

### 4.4. Immunofluorescence Microscopy

BPV-A3 cells grown on a coverslip were exposed to UV, as mentioned, and after 8 h, the cells were fixed in 4% paraformaldehyde and permeabilized with Triton-X-100 (0.1% in PBS–PBST) for 10 min. After 3 washes, the cells were incubated with 2% goat serum in PBST for 1 h and subsequently incubated overnight at 4 °C with phosph-H2AX antibody in 2% goat serum PBST. After 3 washes, cells were incubated with anti-mouse secondary antibody conjugated with Alexa 633 fluor (Thermo Fisher) for 1 h at room temperature, washed, and mounted in Vectashield mounting medium with 4′,6-diamidino-2-phenylindole (DAPI—Vector Laboratories) and analyzed with a Nikon fluorescent microscope.

### 4.5. Phospho-H2AX Immunoblotting

CIN612E cells were pretreated with 2 mM caffeine overnight and exposed to 200 mJ/cm^2^ of UV. Five hours post-irradiation, the cells were lysed in lysis buffer (2% SDS, 150 mM NaCl, 10 mM Tris-HCl, pH 8.0), separated by gradient (4–12%) PAGE, blotted onto an Immobilon-P (Millipore, Burlington, MA, USA) membrane, and probed using phospho (S139) H2AX antibody at a 1:2000 dilution in 2% BSA-TBST (20 mM Tris, 150 mM NaCl, 0.1% Tween-20), and β-actin was used as a loading control.

### 4.6. cDNA Synthesis and Quantitative Reverse-Transcriptase PCR

The CIN612E cell line was treated for 72 h with 2 mM caffeine or PBS control, and total RNA was isolated by TRIzol (Invitrogen, Waltham, MA, USA). Five micrograms of total RNA were reverse-transcribed using Superscript III (Life Technologies, Carlsbad, CA, USA). cDNA was used in the qPCR reactions using the following primers:

HPV31E2—forward: 5′-AGCGTTGTCAGTATCAAAGGC-3′; reverse: 5′-GCTGCATTGTCCAGTCCTCAT-3′. HPV31E1—forward: 5′-ATAGGCGAGCCCAAAAACGA-3′; reverse: 5′-TGGTCTCCAATCTCCCCCTT-3′. HPV31E6—forward: 5′-AAGACCGTTGTGTCCAGAAGA-3′; reverse: 5′-CTGTCCACCTTCCTCCTATGTT-3′. Human β-actin—forward: 5′-GAGGCACTCTTCCAGCCTTC-3′; reverse: 5′-CGGATGTCCACGTCACACTT-3′.

### 4.7. Copy Number of HPV31 in CIN612E Cells

The CIN612E cell lines were treated for 72 h with 2 mM caffeine or PBS control, and genomic DNA was isolated by the phenol-chloroform method. The copy number of HPV31 was measured [29] using HPV31 LCR4 (targeting the locus control region) primer sets [31] and was normalized to the human actin gene.

### 4.8. E2 Transcriptional Activation Assay

The luciferase-based transcriptional activity of E2 was analyzed (DeSmet et al., 2016). Briefly, 20,000 C33a cells were seeded per well on a 96-well plate. The cells were transfected with 10 ng pCG-BPV-1 E2 and 75 ng pGL2-E2BS-Luc using lipofectamine, along with 2 mM caffeine or PBS control. The firefly luciferase activity was measured after 48 h of incubation.

### 4.9. Chromatin Immunoprecipitation

The CIN612E cells were pretreated with 1 mM caffeine/PBS and arrested overnight with 1.5 mM thymidine in 1 mM caffeine/PBS containing E-media. The next day, the cells were released to thymidine-free E-media containing 1 mM caffeine/PBS. After 8 h, a second thymidine treatment was carried out with or without 1 mM caffeine overnight, and double-thymidine-arrested cells were cross-linked with 1% paraformaldehyde for 10 min. The cross-linking was stopped by treating the cells with 0.125M glycine for 10 min. The cells were washed in PBS, and ChIP was performed [29] with HPV31-specific E1 and E2 antibodies [31], along with the control EE antibody. The HPV31-LCR4-primer-set-derived amplification was normalized to 2% input DNA.

### 4.10. Suspension-Mediated Infection (SMI) of PsV and QsV in N/TERT Cells

HPV31Neo QsV and mCherry PsV particles were generated and titrated, and N/TERT cells were infected using the SMI method [20,30]. Briefly, the N/TERT cells were pretreated overnight with PBS or 2 mM caffeine. The cells were then trypsinized, mixed with PsV alone or PsV-QsV combined viruses in F-media, and placed in a 60 mm dish for 8 h in PBS/caffeine. After incubation, the infection media was replaced with K-SFM media for 72 h, and the cells were further grown in PBS/2 mM caffeine, whereafter DNA was extracted using DNeasy Blood & Tissue Kits (Qiagen, Hilden, Germany). The episome copy number was measured as described in [20]. To establish the effect of DDR inhibition on the virus entry, the N/TERT cells were infected with PsV expressing an mCherry reporter gene in the absence or presence of 2 mM caffeine. After 72 h, the cells were visualized using a Nikon fluorescent microscope, and the red mCherry-expressing fluorescent cells were plotted as a percentage of the total cells. For the replication after infection, 100 MOI equivalents of mCherry PsV and HPV31Neo QsV were mixed, and the N/TERT cells were infected. To measure the infection/replication of PsV/QsV, qPCR was carried out using the HPV31 LCR4 primer set and mCherry primers (mCherry-qF2: 5′-AAGGGCGAGGAGGATAACAT-3′; mCherry-qR2: 5′-ACATGAACTGAGGGGACAGG-3′). Human β-actin was used as the internal control. For the fold change calculations, after normalization to the internal control, the HPV31 qPCR signal was divided by mCherry-specific amplicons and represented as HPV31LCR/mCherry.

### 4.11. Statistical Analysis

All the experiments were repeated at least three independent times except when stated otherwise, and the results were averaged. The bar indicates the standard error of the mean (SEM). Student’s t-test was used to identify the significant value.
